# Seroprevalence of *Strongyloides stercoralis* Infection and Its Risk Factors in Pregnant Women Referred to Al‐Zahra Hospital in Guilan Province, North Part of Iran: A Cross‐Sectional Study From 2024 to 2025

**DOI:** 10.1155/japr/9933423

**Published:** 2026-02-12

**Authors:** Mahdieh Sorouri Majd, Eshrat Beigom Kia, Seyedeh Hajar Sharami, Mehdi Mohebali, Azadeh Jafari, Roya Latifi, Zohreh Fakhrieh-Kashan

**Affiliations:** ^1^ Department of Medical Parasitology and Mycology, School of Public Health, Tehran University of Medical Sciences, Tehran, Iran, tums.ac.ir; ^2^ Reproductive Health Research Center, Department of Obstetrics & Gynecology, Al-Zahra Hospital, School of Medicine, Guilan University of Medical Sciences, Rasht, Iran, gums.ac.ir; ^3^ Center for Research of Endemic Parasites of Iran (CREPI), Tehran University of Medical Sciences, Tehran, Iran, tums.ac.ir; ^4^ Department of Pathology, School of Medicine, Tehran University of Medical Sciences, Tehran, Iran, tums.ac.ir

**Keywords:** ELISA, Iran, parasitological methods, pregnant women, risk factors, strongyloidiasis

## Abstract

**Background:**

In immunocompromised individuals, strongyloidiasis may progress to hyperinfection syndrome or disseminated disease. To date, the death of a pregnant woman infected with *Strongyloides stercoralis* has been reported. Chronic cases carry a risk of malnutrition, especially among vulnerable populations like pregnant women and children. Pregnancy‐related immunosuppression increases susceptibility to severe strongyloidiasis. This study assesses strongyloidiasis seroprevalence in pregnant women at Al‐Zahra Hospital, Guilan Province.

**Materials and Methods:**

This cross‐sectional study (September 2024 to February 2025) randomly selected 384 pregnant women referred to Al‐Zahra Hospital, Rasht. After consent, blood (all) and permitted stool samples plus symptom questionnaires were collected. Serum was tested for anti–*S. stercoralis* IgG antibodies using ELISA (NovaLisa). Stool underwent parasitological examination (direct smear, formalin–ethyl acetate concentration method, and nutrient agar plate culture [APC]). SPSS v25 analyzed data using Fisher′s exact test.

**Results:**

The study enrolled pregnant women (gestational age: 1–8 months) aged 15–48 years. The seroprevalence of strongyloidiasis (anti–*S. stercoralis* IgG antibodies) was 2.6%. Six of these individuals, who provided stool samples, also tested positive by parasitological examination. No significant associations (*p* > 0.05) were observed between infection and factors including vegetable washing practices, residential location (urban/rural), education level, occupational exposure, soil contact, clinical symptoms, animal contact, or hypereosinophilia. However, a significant association (*p* < 0.05) was identified between strongyloidiasis and underlying conditions, with gestational diabetes present in 30% of patients.

**Conclusion:**

The overlap of gastrointestinal, respiratory, and hematological symptoms with pregnancy contributes to the underdiagnosis of strongyloidiasis. Early respiratory symptoms are hormonally driven, whereas persistent later symptoms suggest infection (50% strongyloidiasis cases here). Preconception *S. stercoralis* serology and hygiene/animal‐contact education are recommended.

## 1. Background

The etiology of human strongyloidiasis encompasses infection with three nematode taxa: *Strongyloides stercoralis* (*S. stercoralis*), *S. fuelleborni* subspecies *fuelleborni*, and *S. fuelleborni* subspecies *kellyi* [1–4]. The World Health Organization (WHO) classifies strongyloidiasis as a Neglected Tropical Disease (NTD), emphasizing its contribution to long‐term disability, growth impairment in children, and pregnancy‐related complications [5–7]. Globally, an estimated 614 million people are infected with this parasite [8]. In Iran, the northern and southern provinces are recognized as endemic areas for *S. stercoralis*, and the prevalence of this parasite is increasing in many developing countries, driven by heightened migration and international travel [9, 10]. Chronic strongyloidiasis is notably associated with malnutrition, disproportionately affecting vulnerable populations such as pregnant women, infants, and children [6, 7].

Helminthic parasitic infections during pregnancy are known to suppress Th1 inflammatory responses while enhancing Th2 responses. Concurrently, hormonal fluctuations significantly influence immune regulation [11]. Specific adaptations of the immune system during pregnancy, including modulation of Th1/Th2 balance and suppression of cellular immunity, protect against fetal rejection while simultaneously increasing susceptibility to parasitic infections such as *S. stercoralis* [12]. The complexity of physiological, hormonal, and immunological conditions during pregnancy directly influences susceptibility to infections, consequently leading to alterations in the immune system that may favor infection with *S. stercoralis* [13, 14].

Corticosteroid therapy is sometimes employed during pregnancy to prevent preterm labor. However, this approach may exacerbate infections in pregnant women with chronic strongyloidiasis. The condition can contribute to maternal malnutrition, potentially leading to intrauterine growth restriction and low birth weight in infants. In recent years, there has been an increase in reported cases of strongyloidiasis among pregnant women, including fatalities associated with corticosteroid use and the subsequent worsening of infections [15, 16]. Furthermore, adverse reactions to anti‐strongyloidiasis treatments during pregnancy have been documented globally [17]. For instance, ivermectin is classified as FDA Pregnancy Category C and is generally avoided during pregnancy due to limited safety data, unless the benefit outweighs the risk [18].

It is critical to note that hyperinfection syndrome and disseminated strongyloidiasis in immunocompromised individuals can be fatal if treatment is delayed. Parasitological methods are commonly used for diagnosing strongyloidiasis. However, the ELISA technique, recognized for its high sensitivity and specificity, has recently been employed in serological studies and is considered an acceptable method for seroprevalence research [1, 19–22]. In Iran, where strongyloidiasis is endemic, pregnant women represent a high‐risk group requiring focused attention due to the risks posed to both mothers and newborns. Despite this, no studies have previously investigated strongyloidiasis in pregnant women in Iran. To address this gap, this study was conducted for the first time to assess its seroprevalence among pregnant women attending Al‐Zahra Hospital in Rasht (Guilan Province).

## 2. Materials and Methods

### 2.1. Timeline and Study Area

This cross‐sectional study was conducted between September 2024 and February 2025. Participants included pregnant women referred to Al‐Zahra Hospital in Rasht, North of Iran.

### 2.2. Data Collection

#### 2.2.1. Sample Collection

Stool samples were collected from cooperating pregnant women, and 3 mL of blood was obtained from those who had not taken any antiparasitic medication in the preceding 3 months, provided written informed consent, and completed a questionnaire capturing demographic data (e.g., age, occupation, and residence) and clinical symptoms (e.g., gastrointestinal, dermatological, and respiratory). Additional questions addressed vegetable washing practices, soil exposure, animal contact, and medication use. Sample collection was conducted using a random sampling approach to ensure representativeness of the study population. The blood samples were centrifuged at 2500 rpm for 5 min and stored at −20°C until serological testing.

#### 2.2.2. Parasitological Tests

Stool samples from cooperating individuals were examined using standardized parasitological techniques, including direct smear, formalin–ethyl acetate concentration, and agar plate culture (APC).

#### 2.2.3. Serological Tests

Serum samples were evaluated for anti–*S. stercoralis* IgG using a commercial ELISA kit (LOT: STRO‐050N; NovaLisa, Germany) based on a recombinant antigen. Optical density (OD) was measured at 450 nm using a BioTek ELx808 ELISA reader. Antibody concentrations were determined following the manufacturer′s protocol, and reference ranges included positive, > 11 NTU; equivocal, 9–11 NTU; and negative, < 9 NTU. Also, positive results were confirmed with two replicate tests exceeding 11 NTU. Noncompliant results were considered invalid, requiring the assay to be repeated.

Equivocal serological results prompted follow‐up contact with patients for repeat blood collection after 3–4 weeks. Noncompliant participants were excluded. Positive tests were double‐checked. Additionally, complete blood count (CBC) results from routine check‐ups were recorded in the questionnaire.

### 2.3. Sample Size

The sampling process in this study was conducted using a sequential sampling method. The sample size was calculated to be *n* = 384, based on an estimated infection prevalence (*p* = 0.1) [23], a margin of error (*d* = 0.03), and the following formula [24, 25]:

n=z2p1−pd2,

where

• *n* = sample size,
• *z* = confidence coefficient,
• *d* = acceptable margin of error, and
• *p* = estimated prevalence of infection.

### 2.4. Statistical Analysis

Laboratory results from ELISA, parasitology tests, and questionnaire data were analyzed using SPSS version 25. Descriptive statistics (e.g., frequency, percentage, and cumulative percentage) and inferential statistics (chi‐square test and *p* value) were employed. The association between strongyloidiasis and risk factors was assessed using Fisher′s exact test.

## 3. Results

### 3.1. Characteristics of the Study Population

This study involved 384 pregnant women aged 15–48 years. Fifty percent had experienced at least two pregnancies, and 25% reported a history of at least one miscarriage. Of these women, 81.3% resided in urban areas and 18.7% in rural areas of Guilan Province. Regarding educational attainment, 80% of participants had a high school education or lower, and 89.8% were homemakers. Additionally, 25.5% had soil contact, and 32% had animal contact. Only 0.3% used disinfectants to wash vegetables. In terms of health status, 54.9% of participants had at least one underlying medical condition, with diabetes being the most common. The most prevalent clinical symptoms were gastrointestinal, including nausea (48.4%), constipation (23.4%), and loss of appetite (22.4%).

### 3.2. Results of Indirect ELISA Testing

**Figure 1:**
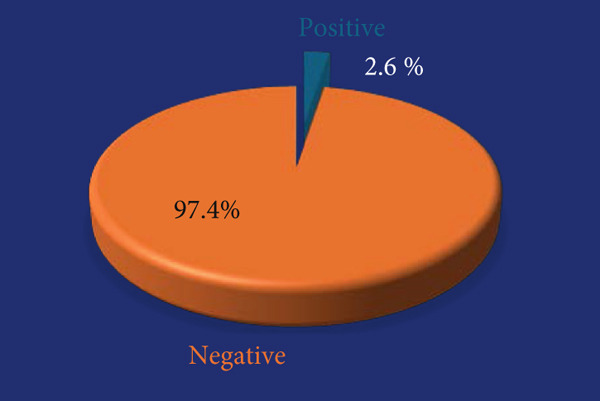
The seroprevalence of anti–*S. stercoralis* IgG antibody in pregnant women in the present study.

The seroprevalence of strongyloidiasis, determined using the commercial NovaLisa kit, was 2.6% (Figure 1).

Of these 10 individuals, three had a serological titer greater than 100 NTU. Of the sample, 90% were urban residents, and all used tap water without disinfectants to wash vegetables. Eighty percent exhibited at least one clinical symptom related to gastrointestinal, respiratory, or dermatological conditions. Thirty percent had diabetes as an underlying condition. Additionally, 40% reported soil contact, and 70% had animal contact Notably, one individual had contact with both dogs and cats, and another had contact solely with cats (Table 1).

**Table 1:** Information on individuals with positive serological test results for anti–*S. stercoralis* IgG antibody.

**Row**	**Age**	**Place of residence**	**Eosinophilia**	**Serological titer (NovaTec unit [NTU])**	**Clinical symptoms**	**Soil contact**	**Animal contact**	**Underlying condition**	**Medication use**	**Method of vegetable washing**	**Parasitology tests (direct smear, formalin–ethyl acetate culture)**
1	37	City	2	40.3	Shortness of breath, abdominal pain, diarrhea	Yes	Yes	Hypertension, hypothyroidism, cancer	Methyldopa, levothyroxine sodium	Tap water without disinfectants	Culture positive
2	36	City	2	34	Pruritus	Yes	Yes	Thrombocytopenia	—	Tap water without disinfectants	Direct smear, formalin–ethyl acetate, culture positive
3	36	City	3	35.4	Nausea, loss of appetite	No	Yes	Hypertension, diabetes	Insulin, methyldopa	Tap water without disinfectants	^a^
4	24	City	2	23.2	—	No	Yes	Depression	—	Tap water without disinfectants	^a^
5	38	City	2	>100	—	Yes	Yes	Diabetes	Insulin	Tap water without disinfectants	Culture positive
6	35	City	5	14.34	Shortness of breath, nausea	No	Yes	Hypothyroidism	Levothyroxine	Tap water without disinfectants	Culture positive
7	32	City	2	16.68	Shortness of breath	No	Yes	—	Pdxane 6000 enoxaparin sodium	Tap water without disinfectants	^a^
8	41	Village	6	>100	Shortness of breath, cardiac condition	No	No	Cardiac disorder, diabetes	Insulin	Tap water without disinfectants	Culture positive
9	22	City	—	>100	Nausea	No	No	Hypothyroidism, cancer	Levothyroxine	Tap water without disinfectants	Culture positive
10	36	City	3	29.52	Shortness of breath, abdominal pain, bloating	Yes	No	Anemia	Iron supplement	Tap water without disinfectants	^a^

^a^Indicating individuals who did not cooperate in providing stool samples.

In the present study, based on the results of the Mann–Whitney *U* test, the age in the seropositive group ranged from 22 to 41 years (mean ± SD: 33.7 ± 6.09), which was higher than that in the seronegative group, with an age range of 15–48 years (mean ± SD: 31.91 ± 6.25). However, no statistically significant difference in age was observed between the two groups (*p* > 0.05).

Based on the results of Fisher′s exact test for this sample size, no significant association was observed between *S. stercoralis* IgG serostatus and education level, occupation, or residence in urban or rural areas, soil contact, animal contact, or washing vegetables with tap water without using disinfectants (*p* > 0.05). Similarly, no significant association was found between *S. stercoralis* IgG serostatus (positive or negative) and gastrointestinal symptoms (nausea, anorexia, diarrhea, constipation, abdominal pain, vomiting, or bloating), dermatological symptoms (urticaria or pruritus), or respiratory symptoms (cough or dyspnea) (*p* > 0.05 for all). Of the 384 participants, 186 exhibited gastrointestinal symptoms, 68 exhibited cutaneous symptoms, and 120 exhibited respiratory symptoms; the details are stratified by seropositive and seronegative status for *S. stercoralis* IgG antibody (Table 2). However, a significant association was identified between the presence of an underlying disease and *S. stercoralis* IgG–positive serology results (*p* = 0.026).

**Table 2:** Comparison of clinical symptoms: *S. stercoralis* IgG–positive vs. *S. stercoralis* IgG–negative groups.

**Clinical symptoms**	***S. stercoralis* IgG–seronegative individuals (** **n** **, %)**	***S. stercoralis* IgG–seropositive individuals (** **n** **, %)**
Respiratory	116, 31.01%	4, 40%
Cutaneous	67, 17.9%	1, 10%
Gastrointestinal	181, 48.39%	5, 50%

Regarding the association between indirect ELISA test results and hematological parameters, the white blood cell (WBC) count in the seropositive strongyloidiasis group was lower than that in the seronegative group, and the platelet (PLT) count in the seropositive group was significantly lower than that in the seronegative group. Twenty‐one individuals exhibited eosinophilia above 5%, of whom only two tested positive for *S. stercoralis* IgG antibody. Based on statistical analysis, no significant association was observed between *S. stercoralis* IgG serology results and the percentage of hypereosinophilia (*p* > 0.05) (Table 3).

**Table 3:** Hematological test results in anti‐*S. stercoralis* IgG‐positive and IgG‐negative patients.

**Serology**			**WBC**	**RBC**	**Hb**	**PLT**	**EOS**
Positive	N	Valid	10	10	10	10	10
		Missing	0	0	0	0	0
	Mean		10.080	4.0420	11.0300	180.90	2.80
	Median		9.100	4.0850	10.8000	176.50	2.00
	Std. deviation		2.5594	0.46609	1.12847	62.410	1.549
	Minimum		7.1	3.42	9.40	90	1
	Maximum		15.0	4.90	12.90	294	6

Negative	N	Valid	370	370	370	370	371
		Missing	4	4	4	4	3
	Mean		10.617	4.1159	11.7041	217.04	2.17
	Median		10.000	4.0150	11.7000	210.00	2.00
	Std. deviation		3.2831	1.52545	1.44162	65.055	1.788
	Minimum		4.4	2.72	3.52	76	0
	Maximum		32.0	32.00	16.60	460	28

### 3.3. Parasitological Test Results

Given that stool sample collection was not mandatory and was performed only with the participants′ cooperation and consent—considering their status as pregnant women—samples were obtained from only 207 out of 384 individuals. Among these 207 participants, six were diagnosed with strongyloidiasis by parasitological methods and also tested positive for antibodies against *S. stercoralis*. Of these six cases, five were confirmed positive by the APC method. One individual tested positive by all three methods. Culture media were retained for up to 1 week and examined daily from 24 h post inoculation until the end of the seventh day. In most cases, cultures from seropositive participants turned positive between Days 2 and 3. A culture was considered positive if characteristic larval tracks or the presence of larvae was observed in the medium. Larvae were then morphologically identified as *S. stercoralis* [26].

### 3.4. Treatment of Patients

The laboratory results were sent to the patients, who were advised to consult their physicians. Due to the potential adverse effects of antiparasitic medications on the fetus, obstetricians preferred that treatment be postponed until after delivery, with patients instructed to return to the laboratory for repeat parasitological examinations 4 weeks following the completion of treatment.

## 4. Discussion

Strongyloidiasis is recognized as a NTD [1], with its control and elimination in endemic regions by 2030 being a key objective of the WHO [5]. In individuals with intact immune systems, strongyloidiasis is often asymptomatic. The most severe forms of *S. stercoralis* infection include hyperinfection syndrome and disseminated disease, which typically occur in immunocompromised individuals [27]. Immunomodulation during pregnancy can attenuate the host defense against intracellular pathogens. This state increases susceptibility to various viral and parasitic infections [28–30].

In the present study, 384 pregnant women aged 15–48 years were examined in Rasht, where a 2.6% seroprevalence of strongyloidiasis was observed. Based on Fisher′s exact test, no significant associations were found between *S. stercoralis* serostatus and participants′ educational level, occupation, or place of residence (urban or rural) and age (*p* > 0.05).

However, a significant association was identified between the presence of an underlying condition and anti‐*S. stercoralis* IgG seropositivity (*p* = 0.026). In this study, 53 participants had diabetes, of whom only eight (2.1%) were diagnosed with pregestational diabetes. Of the 10 individuals with strongyloidiasis, three individuals (30%) had gestational diabetes. Among the 50 seronegative diabetic participants, eight individuals (16%) had pre‐existing gestational diabetes, whereas 42 individuals (84%) had gestational diabetes mellitus. Given that this is the first study conducted in pregnant women and no prior research has specifically evaluated the association between gestational diabetes and strongyloidiasis, the sample size in the present study precludes definitive conclusions regarding a potential link between gestational diabetes and *S. stercoralis* infection. However, previous studies have reported an association between Type 2 diabetes and *S. stercoralis* infection [19]. Further investigations are required to confirm whether gestational diabetes may be associated with *S. stercoralis* infection.

Gastrointestinal issues are among the most common complaints during pregnancy, influenced by hormonal changes that affect the digestive system. Many pregnant women experience changes in appetite, such as increased hunger, reduced appetite, food cravings, aversions, nausea, and vomiting [31]. In this study, most participants reported at least one gastrointestinal symptom, owing to the considerable overlap between the clinical manifestations of pregnancy and strongyloidiasis, which complicated the differentiation of strongyloidiasis specific symptoms. Among the seronegative participants, gastrointestinal symptoms were reported with the following prevalences: abdominal pain in 15.5%, bloating in 7.2%, vomiting in 3.47%, constipation in 24.06%, diarrhea in 3.47%, nausea in 0.8%, and anorexia or loss of appetite in 22.9% (Table 2).

The most frequently reported clinical symptoms in this study were gastrointestinal, including (in order of prevalence) nausea, anorexia, diarrhea, constipation, abdominal pain, vomiting, and bloating, followed by respiratory symptoms (cough and dyspnea) and dermatological symptoms (urticaria and pruritus). These findings align with those reported by Sari et al. [22]. However, some studies in Iran [26, 32, 33] have reported a higher prevalence of dermatological symptoms compared to respiratory symptoms. Notably, neither the present study nor the referenced studies reported cases of larva currens.

Hypereosinophilia is defined as an eosinophil count exceeding 5000 cells/*μ*L [34]. Statistical analysis showed no significant association between anti–*S. stercoralis* IgG seropositivity and hypereosinophilia (*p* > 0.05). In contrast, Sharifdini et al. [26] reported eosinophilia with a mean of 22.7% in 51 patients, with a significant association between increasing age and reduced eosinophilia. In the present study, medical records of patients with strongyloidiasis revealed eosinophilia ranging from 1% to 6% with overall eosinophilia in the study population varying between 1% and 28%. Twenty‐one participants exhibited eosinophilia greater than 5%, of whom only two tested positive for strongyloidiasis serologically, while the remaining 19 were seronegative for strongyloidiasis. Given the prevalence of other parasitic infections in this region, such as fascioliasis and toxocariasis, further investigations are warranted to determine the underlying causes of hypereosinophilia.

No significant associations were observed between anti–*S. stercoralis* IgG seropositivity and contact with soil, animals, or washing vegetables with tap water without disinfectants (*p* > 0.05). Similarly, Sari et al. [22] found no association between vegetable washing practices and strongyloidiasis but reported a significant association with dog contact (*p* < 0.05). In the present study, one patient had contact with both dogs and cats and another with cats only. Genetic studies on the *Cox*1 gene of *S. stercoralis* have identified similarities between isolates from humans, dogs, and cats [35, 36], highlighting the zoonotic potential of this parasite and underscoring the need for further research in endemic regions.

Dyspnea during the first trimester of pregnancy is often attributed to elevated progesterone levels, which affect the respiratory system, increasing the frequency and depth of breathing to meet the oxygen demands of the fetus. In the third trimester, dyspnea may result from fetal growth exerting pressure on the diaphragm, though this typically diminishes after week 36 as the fetus descends. However, severe respiratory symptoms in the second and third trimesters are not hormonally driven and warrant investigation for infectious or other respiratory conditions. Studies indicate that approximately 50% of pregnant women experience dyspnea during physical activity, and 20% report dyspnea at rest [37], contributing to the overlap of strongyloidiasis‐related respiratory symptoms with those in healthy pregnant women. In this study, 50% of individuals with strongyloidiasis experienced severe respiratory issues, with one case in the second trimester and four in the third trimester, suggesting that strongyloidiasis‐related respiratory complications in pregnant women in endemic areas merit further attention. Pregnant women residing in regions endemic for *S. stercoralis* who present with excessive respiratory symptoms from the early months of pregnancy or with recurrent intermittent gastrointestinal complaints that exceed typical pregnancy‐related symptoms in severity and persistence, particularly in the presence of hypereosinophilia, should raise clinical suspicion of an underlying parasitic infection, especially strongyloidiasis, in these endemic areas.

The findings of our study highlight the challenges associated with parasitological diagnosis of *S. stercoralis*, particularly in patients with constipation, which limited their ability to provide multiple fecal samples, with most participants submitting only a single sample. This aligns with previous research indicating that single fecal examinations yield low sensitivity, ranging from 6% to 10% for *S. stercoralis* detection, as demonstrated by Knopp et al. [38], who reported an increase in sensitivity with multiple examinations. However, the requirement for multiple samples and skilled technicians poses significant limitations, especially in resource‐constrained settings. In contrast, serological methods, such as the NovaLisa *Strongyloides* ELISA kit used in our study, which employs the recombinant NIE antigen derived from the third‐stage larva of *S. stercoralis* and exhibits no cross‐reactivity, offer a promising alternative. Immunological techniques, including ELISA and luciferase immunoprecipitation systems (LIPS‐NIE), have demonstrated higher sensitivity and specificity, thereby reducing diagnostic time and mitigating challenges associated with intermittent larval output [39–41]. Given these advantages, serological testing is recommended as a primary screening tool, particularly in prepregnancy assessments, to enhance diagnostic accuracy and improve patient outcomes in populations with low compliance for repeated parasitological sampling.

## 5. Limitations

This study identified only 10 positive serum samples out of 384 participants, which significantly reduced the statistical power for subgroup analyses and the investigation of associations with risk factors. Consequently, the generalizability of the findings is limited due to the small number of positive cases.

Another limitation was the incomplete participation of pregnant women in providing stool samples (or multiple stool specimens). Many were unable or unwilling to cooperate because of pregnancy‐related constipation and the lack of suitable conditions for sample collection.

Furthermore, as the study was conducted as part of a master′s thesis with a fixed and limited budget, it was not feasible to include a larger sample size.

## 6. Conclusion

The overlap between the clinical manifestations of strongyloidiasis and common pregnancy‐related complaints, including gastrointestinal and hematological disturbances, probably contributes to the underdiagnosis of *S. stercoralis* infection in pregnant women. The observed association with gestational diabetes may serve as a potential indicator of *S. stercoralis* infection and merits further investigation. The lower prevalence of infection in pregnant women is likely attributable to heightened self‐care practices and the exclusion of higher risk occupations and male participants compared with previous general‐population studies, further underscoring the role of behavioral and demographic factors in disease transmission. Although our findings suggest that serological screening for *S. stercoralis* could be valuable in preconception care, complemented by reinforced hygiene education, such recommendations remain preliminary. Larger, multicenter studies are needed to confirm these observations, evaluate their generalizability, and develop evidence‐based guidelines for screening and management in pregnant populations.

**Nomenclature**
ELISA: enzyme‐linked immunosorbent assay
WHO: World Health Organization
IgG: immunoglobulin G
CBC: complete blood count
APC: agar plate culture
NTU: NovaTec units (used in the context of the Tec Nova ELISA kit for antibody)
OD: optical density
WBC: white blood cell count
PLT: platelet count
PIBF: progesterone‐induced blocking factor
Th1: T‐helper 1 (immune response type)
Th2: T‐helper 2 (immune response type)
IL‐4: Interleukin‐4
IL‐10: Interleukin‐10
IL‐5: Interleukin‐5
TNF‐*α*
: tumor necrosis factor‐alpha
Treg: regulatory T cell
SPSS: Statistical Package for the Social Sciences

## Ethics Statement

The present study was approved by the Ethics Committee of the School of Public Health, Tehran University of Medical Sciences, under code IR.TUMS.SPH.REC.1403.162. Signed consent was received from all study participants, and their information was kept confidential. All methods were done according to relevant guidelines and regulations.

## Consent

All participants provided written informed consent for the publication of the study findings. No identifiable personal data or images of participants are included in this publication.

## Disclosure

All authors evaluated and endorsed the final manuscript, taking responsibility for the accuracy and integrity of the data presented.

## Conflicts of Interest

The authors declare no conflicts of interest.

## Funding

This study was funded by the Tehran University of Medical Sciences and Health Services, IR.TUMS.SPH.REC.1403.162.

## Data Availability

The data underlying the results of this study can be obtained from the corresponding author upon reasonable request.
